# Design Criteria of Soft Exogloves for Hand Rehabilitation-Assistance Tasks

**DOI:** 10.1155/2020/2724783

**Published:** 2020-07-20

**Authors:** Juana-Mariel Dávila-Vilchis, Juan C. Ávila-Vilchis, Adriana H. Vilchis-González, LAZ-Avilés

**Affiliations:** ^1^Faculty of Engineering, Universidad Autónoma del Estado de México, Toluca 50130, Mexico; ^2^Cátedras CONACYT, Universidad Autónoma del Estado de México, Toluca 50130, Mexico

## Abstract

This paper establishes design criteria for soft exogloves (SEG) to be used as rehabilitation or assistance devices. This research consists in identifying, selecting, and grouping SEG features based on the analysis of 91 systems that have been proposed during the last decade. Thus, function, mobility, and usability criteria are defined and explicitly discussed to highlight SEG design guidelines. Additionally, this study provides a detailed description of each system that was analysed including application, functional task, palm design, actuation type, assistance mode, degrees of freedom (DOF), target fingers, motions, material, weight, force, pressure (only for fluids), control strategy, and assessment. Such characteristics have been reported according to specific design methodologies and operating principles. Technological trends are contemplated in this contribution with emphasis on SEG design opportunity areas. In this review, suggestions, limitations, and implications are also discussed in order to enhance future SEG developments aimed at stroke survivors or people with hand disabilities.

## 1. Introduction

Hand and finger motions are imperative for grasping and manipulation tasks. Nonetheless, people who have suffered from cerebral palsy (CP), stroke, or spinal cord injury (SCI) have great difficulty in accomplishing these activities of daily living (ADL) by themselves. A person with any of these pathologies could present clenched fist, spasticity, uncoordinated motions, loss of strength, or diminished dexterity. These are consequences of a neuronal impairment that is responsible for controlling motricity, muscle endurance, and tonicity [1]. Worldwide, more than 15 million people are affected each year [2], and only 11.6% of the stroke survivors are able to recover dexterity [3]. Patients with these disabilities can, freely, flex their hand muscles but show abnormal resistance when extending them [4], requiring physical rehabilitation or assistance.

Other hand motor deficits are caused by ageing or hand deformities such as rheumatoid arthritis or osteoarthritis, because cartilage weakens, muscle mass decreases, and joint stiffness increases [5]. More than 50 million elderly people have difficulties to achieve accurate gripping and pinching forces, and their range of motion (ROM) is limited as well as their work area [6].

Therefore, people with hand disabilities can initiate a prompt rehabilitation protocol in order to start recovering motor skills, stop joint stiffness, and increase their independence and self-esteem [7]. Physical and occupational therapies are the most common treatments to recover patients' movements, for example, adduction-abduction or flexion-extension of finger, wrist, or elbow joints. However, these routines can be exhausting, time-consuming, and, relatively, costly since patients require the assistance of a therapist whose availability is uncertain [8].

Normally, rehabilitation programs are customized for each patient due to their impairment, age, and anthropometric dimensions. Moreover, these robot procedures are classified into three main assistance levels: passive assisted mode (PAM), active assisted mode (AAM), and active resistive mode (ARM) depending on the recovery status of patients and support of a robot [9].

Literature has reported that rehabilitation protocols can be executed by robots or soft wearable devices which have emerged as a therapy tool with safe human interactions, low weight, and affordable systems [10]. Particularly, SEG have become an alternative approach in the effort to overcome hand dysfunctions and assist patients with handling tasks. SEG have the ability to combine conventional therapy with wearable systems to mimic the natural movement of fingers in order to increase their mobility, preventing spasticity and joint stiffness [11].

SEG have mainly evolved in terms of their design, fabrication, and control [12]. Pioneering designs started using sport gloves incorporating a control system [13, 14]. Then, SEG proposals explored synthetic leather [15], rubber [16, 17], and fabrics [18, 19] to provide flexible human-robotic interactions as in the case of bike gloves [20]. Elastomers have become the primary option to empower flexibility and lightness [21]. Moreover, instead of closed palm designs (CPD) where the whole hand is covered with the glove, open palm designs (OPD) with bare hands use elastomers trying to behave as a natural extension of the human hand to compete with skin properties in order to achieve a suitable contact with objects [22, 23]. Other assistance SEG have been developed for material handling in hazardous environments, support in heavy-lifting tasks [24, 25], or extravehicular tasks in space [26].

Mostly, SEG systems have been driven by electrical energy or fluid (pneumatic or hydraulic) pressurization. Regarding electrical power supplies, tendon-driven systems employ linear actuators to push and pull cables embedded in Teflon tubes [27]. Pneumatic actuation includes fiber-reinforced elastomer actuators (FREAs), inflatable chambers, or pneumatic artificial muscles, commonly known as McKibben muscles [28].

People with hand dysfunctions demand for reliable SEG to improve their quality of life. Nevertheless, the lack of affordable and accessible SEG for hand impairment patients with low-cost manufacturing processes is still a significant challenge. Therefore, this paper has reviewed the progress in the field of SEG for neuromuscular rehabilitation and assistance to overcome hand motor dysfunctions.

The main contribution of this paper is the identification and classification of 13 design criteria to provide a set of guidelines for SEG developments based on an extensive review of the state of the art and of the technique from the last decade. Moreover, a detailed description of 91 SEG systems is provided along with implications, limitations, and suggestions for future developments.

This paper is organized as follows.  Section 2 presents, classifies, and discusses the criteria that are proposed for SEG design based on reported devices and specific literature.  Section 3 reports SEG's development guidelines together with the characteristics of the 91 reviewed devices.  Section 4 provides a discussion concerning significant aspects (limitations, implications, and suggestions) to be taken into account for future developments of SEG systems. Conclusions are at the end of this document in Section 5.

## 2. SEG Design Criteria

Hand mobility characterization in SEG designs has turned out to be a challenge since hand anatomy is one of the most complex kinematics parts of the human body with 20 DOF for the whole of the fingers: one for abduction-adduction in every finger (thumb included); 12 for flexion-extension for index, middle, ring, and pinkie fingers; and three for thumb including opponent motion [29].

**Figure 1:**
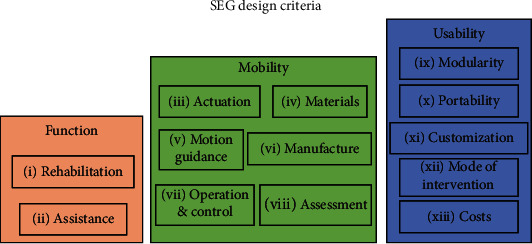
Classification of soft exo-gloves design criteria.

In this paper, 2 function criteria, 6 mobility criteria, and 5 usability criteria are proposed in order to enhance SEG designs and enable fast developments. These design criteria are based on the aspects that have been identified from the 91 SEG systems reported in this article and on the soft wearable device's methodology established in [28].  Figure 1 illustrates the proposed criterion classification.

Moreover, biological inspiration has come to the fore in SEG design to emulate an animal's motion looking for stability [30] or optimal grasping tasks [31]. According to [8], SEG should weigh less than 500 g, provide easy and comfortable donn-doff, and achieve 10 open-close finger cycles per minute for effective actuation. Regarding SEG mechanical design, authors in [29] suggest taking into account the number of joints and working DOF, the type of actuators, and the application. Other attributes in SEG design should adopt the characteristics of a rehabilitation device which include mode of intervention (unilateral or bilateral), number of DOF, target portion (distal, proximal, or quantity), and motion guidance (passive or active), among others [32].

Based on reported literature, the next paragraphs discuss the criteria presented in Figure 1.

### 2.1. SEG Function Criteria

SEG are classified into rehabilitative or assistive devices depending on their purpose [14]. SEG systems must be able to execute physical therapy and manipulation tasks to offer efficient and competitive devices for those with hand disabilities. Then, rehabilitation and assistance criteria must consider the aspects discussed in the respective paragraphs.

#### 2.1.1. Rehabilitation Criterion

Rehabilitation SEG are designed to help the patient regain strength, dexterity, and coordination to recover hand functionality and range of motion (ROM) [33]. These SEG are focused on performing specific fist motions such as full, hook, straight, and tabletop [18] or open-close to improve grasping tasks [34].

Thumb, index, and middle finger flexion-extension is needed for strong grasping [31, 35]. Supplementary motions such as adduction-abduction are required to grasp and release objects in a more natural way [36]. Furthermore, flexion at the interphalangeal (IP) and metacarpophalangeal (MCP) joints with rotation at the carpometacarpal (CMC) joint is necessary to reproduce thumb opposition [37]. Other SEG are able to perform wrist flexion [38], wrist radial-ulnar deviation [16], or forearm pronation-supination motion [39].

SEG rehabilitation routines can include virtual reality in order to analyse the effects of brain stimulation when executing specific tasks [40]. Patients are immersed in a game environment where they achieve manipulation tasks such as squeezing oranges, catching butterflies, or grabbing objects [39]. Other SEG rely on neuroimaging techniques [41] or provide feedback to assess a patient's conditions and monitor their progress [42]. Nevertheless, it is not enough to train the brain and do physical therapy; a successful rehabilitation process depends on the patient's response and their own capabilities [43].

Depending on each rehabilitation protocol, the required time to use a soft exoglove varies. For instance, 60 minutes per day is recommended by [44]. Pilot tests performed by [45] suggested rehabilitation sessions from 30 to 40 minutes 5 days a week. Authors in [38] recommend 45 minutes but no more than 90 minutes per day to avoid SEG strain deformations. Authors in [46] suggest 180 minutes per week, while authors in [39] determine that 30 minutes per day over the course of 20 sessions is necessary for a positive sizable impact on the impaired hand. Furthermore, to achieve a successful rehabilitation program, patients should combine 30 minutes of SEG training with 30 minutes on occupational therapy per day [47].

#### 2.1.2. Assistance Criterion

Eating, dressing, and writing are everyday actions that are done unconsciously. Nevertheless, those tasks turn out to be a tough challenge for people with hand dysfunctions. Normally, patients depend on their family or on a therapist to assist them [48]. Hence, assistive SEG are intended to help patients to achieve manipulation tasks despite their restricted ROM, to interact with their surroundings, and to execute ADL by themselves. These systems are recommended when rehabilitative SEG are not enough to overcome patient stiffness [49].

SEG for assistance tasks are designed to perform three integral functions of the human hand: (i) finger mobilization, (ii) holding (grasping and gripping) with high precision and strength, and (iii) manipulation for positioning and releasing objects [8]. Assistive SEG should execute grasping, holding-lifting, and releasing motions as continuous actions to achieve a complete manipulation [50]. To achieve stable grasping, thumb, index, and middle fingers must be included on SEG systems [35]. According to [51], soft exoglove devices should provide 8 N of grasping force to manipulate an object with a mass of 1.5 kg.

### 2.2. SEG Mobility Criteria

From a functional perspective, authors in [52] propose that weight, size, and power consumption can define an efficient soft exoglove that fits the anatomical ROM of the human hand. The mass of the whole system should not exceed 3 kg to be considered as an assistive device [50]. These characteristics are included in criteria 3 to 8 (see Figure 1): actuation, materials, guidance mode, manufacture, operation and control, and assessment that are discussed as follows.

#### 2.2.1. Actuation Criterion

As aforementioned, tendon-driven actuators use wires to emulate human tendon functions as flexion-extension motion. This type of actuation can include Bowden cable transmissions to separate the control unit from the end effector and reduce weight [53]. Also, artificial muscle wires have been proposed to avoid friction [54], and shape memory alloys (SMA) have been employed due to their elasticity [55] and high force-weight ratio [26].

On the other hand, pneumatic actuators could be embedded into inflatable air bladders [16] and into a double layer sheet with curved rubber muscles [15] or made of flexible electrostatic discharge plastic sheet materials [1, 56]. The McKibben muscles represent an affordable choice [57] and have the ability to constrain any radial expansion during pressurization [58]. Hydraulic actuators offer high load capacity [11].

A new trend is hybrid actuation which fits hand motion shape using soft pneumatic actuators and tendon-driven operation [7], providing customization based on rigid frames and soft muscles [48].  Table 1 reports the advantages and disadvantages of different SEG actuations.

When using a soft glove, patient safety must be guaranteed. Thus, all SEG must include different safety strategies and levels in their design. For example, on cable actuation, mechanical stops, torque, or tension limiters have been implemented [59]. Regarding pneumatic actuation, solenoid and exhaust valves are employed along with pressure regulators to control air flow or avoid air returns [41]. Quasistatic, dynamic, and material failures are discussed in [60], where measures that can be considered in order to avoid unsafe situations for soft robots are provided.

Other safety levels have been applied to the electrical configuration such as emergency stops, watch dogs, or physical decoupling of power interfaces from logic ones by electromagnetic couplings [51]. In addition, by using closed-loop control (CLC) schemes, sensing errors are minimized and operation in a stable regime is ensured to avoid hyperextension at the wrist or overflexed fingers, for instance [20]. At the programming level, haptic feedback is also included to prevent accidents [61].

More specialized safety strategies related to robots can be considered, such as safety standards or means to guarantee system dependability [62] as fault prevention, fault removal, fault forecasting, and fault tolerance [63]. Being safety a priority aspect, it constitutes a current research area by itself and must be taken into account in the development of SEG systems. Concerning rehabilitation robots, ISO-IEC 80601-2-78 must be taken into account. Many specialized documents are recommended for readers interested in this topic and for researchers and engineers working in SEG design (see, for instance, [64–66]).

Additionally, relevant features for actuators have been identified in SEG literature or proposed in this paper. For instance, current developments have focused on improving actuator design to tackle more DOF [67]. During SEG assembly, the actuators are mounted into the dorsal side of the hand to avoid finger movement obstruction [68] and can be removed from the glove [69]. Actuators must not affect the active ROM of the finger joints and should allow free motions with more contact area for grasping tasks in a compliant manner [21].

Furthermore, actuators should take less than 4 s for full grasping [1]. The length of actuators should not be longer than the length of the fingers to avoid mismatching problems between them [23]. Actuators with low power consumption and continuous hours of operation are recommended.

#### 2.2.2. Material Criterion

To enhance SEG operation, researchers continue to seek compliant, flexible, and lightweight materials to easily conform hand-finger anatomy with the shape of an object [41]. Hence, the payload capacity of elastomers has been exploited to obtain an elastic modulus similar to that of human tissues and avoid cumbersome designs [70].

Nonferromagnetic materials such as nylon, neoprene, polyester, or synthetic leather have been selected as compliant and affordable options to increase conformability and grip strength and reduce pressure on the skin [51, 71]. Additionally, silicon materials offer stable fastening and prevent slippage [72]. These synthetic polymers are easy to wash and do not absorb sweat compared to textile materials [23].

SEG made of fabrics have low cost and offer minimal mechanical impedance to finger motion [73]. Hence, coated fabric SEG systems with thermoplastic polyurethane (TPU) actuators are recommended for customization and to avoid slipping or muscle expansion problems [74].

Actuators made of fabrics work at lower pressures than elastomer actuators due to their inherent stiffness [75]. Therefore, several researchers have work on design, characterization, manufacture, and evaluation of soft elastomer actuators for hand [76–78] and wrist [79] rehabilitation.

To match and support finger flexion-extension, some designs include multisegment elastomers with fiber reinforcement [80, 81] or corrugated fabric layers [41, 43] which are pressurized from 70 kPa up to 375 kPa [75]. Other designs include rigid plastic hoops [67] or nylon strings [82] to avoid radial deformations in FREA.

Material selection has also played a significant role in fastening the actuators to the glove or fingers in a safe way. Mostly, SEG proposals have employed magnets [83] or straps made of Velcro® [8, 18], fabrics [84], and rubber [24]. Other designs had opted for sewing the components [71] or separating the system from the actuators to reduce weight. Actuators can be attached to the wrist through elastomer bracelets [39] or synthetic hide covers [25, 31].

#### 2.2.3. Manufacture Criterion

Mobility is also determined by manufacturing processes since specific elements can be obtained by particular methods that, additionally, can determine the weight and dexterity of the system. Conventional manufacturing procedures involve polymer casting molds [85], reinforcements and inclusions [11], additive manufacturing, thin-film manufacturing, shape deposition manufacturing, and bonding [86].

Mostly, 3D printing two-part mold has been employed for SEG spacers [23], cable guides [73], and elastomer actuators [87] where one mold is used to create a fluid chamber inside the actuators and the second one is addressed to create a fabric layer on top of the actuators [41, 43]. Nevertheless, low repeatability is the main drawback during this process [48].

Recent developments involve thermomethods [34], inverse flow injection [42, 82], lost wax molding [88], or fused deposition modeling with 3D printing at home to reduce SEG costs and facilitate its acquisition [89]. However, there is still room to improve SEG materials and fabrication with low costs.

New trends are oriented to hybrid designs where they combine rigid and soft components to obtain more hand poses and more DOF [90] and provide active training that encourages user participation [91].

#### 2.2.4. Motion Guidance Criterion

SEG are designed to follow specific trajectories defined by a therapist depending on the impairment of the patient. These trajectories seek to achieve a functional ROM during both active and passive modes.

SEG is aimed at promoting active finger flexion and passive extension to increase patient autonomy during eating or drinking tasks [46, 92]. In the active assistance mode, patients attempt to move their hand and SEG are an additional aid to complete the desired ROM [93] whereas in the passive assistance mode, the exoglove provides all the assistance to guide the desired movement [94]. In the patients' force recovery processes, effective SEG systems should, actively, participate with intensive training based on active and repetitive practical motions [95].

SEG should combine active and passive mobilizations for successful hand rehabilitation. For example, authors in [15] provide active extension on each finger. In [8], SEG also exert passive extension with active flexion and thumb opposition for grasping tasks. Other systems include active finger adduction-abduction [85] and perform flexion-extension motions [39, 71]. More sophisticated SEG systems have already begun an age that allows patients to perform a desired movement. When patients are able to achieve functional ROM, the system will have no effect on the hand [41] or will create an opposite force to improve the power of the patient.

**Figure 2:**
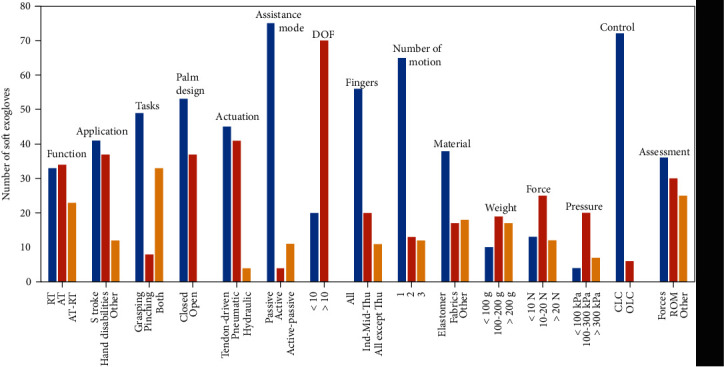
Frequency of SEG aspects reported in Table 3.

Most of the reported SEG systems focus on PAM, a few on AAM as well as on the combination of active and passive modes (see Figure 2).

#### 2.2.5. Operation and Control Criterion

SEG operation is defined by their type of actuation and their components. Tendon-driven wires require servomotors, gearboxes, spools, and force/torque sensors to move them. Pneumatic systems require a compressor, electrovalves or proportional valves, pressure sensors, or regulators. All these components are controlled on a data acquisition board which is plugged to a PC or uses Bluetooth as a communication interface for the SEG system [74].

Different schemes have been proposed to operate and control SEG systems; for example, in [14], Faulhaber 1226 006B motors, CompactRIO board, and LabVIEW® are used. Authors in [21] use DCX22 motors, a control board TMS320F2808®, and Simulink®. Additionally, graphical user interfaces (GUI)® have been implemented as a communication channel for SEG systems [88, 91]. A broad range of operation and control possibilities exists to select microcontrollers and interfaces relying on desired real-time response, accuracy, number of components involved in the operation and control strategies, and specific requirements of each SEG system.

Normally, open-loop control (OLC) and closed-loop control strategies are implemented during SEG operation. OLC schemes have used springs [1, 34] or mechanical switches [54] for manual operation where patients are able to drive an actuator to accomplish a specific task [96]. OLC strategies require the system to be stable by construction. To regulate the desired variables or to track specific trajectories that ensure patient safety while using a soft exoglove, CLC strategies are implemented [97]. To achieve acceptable motions in CLC schemes, sensors are directly attached to SEG [98, 99] without the patient worrying about making accurate movements.

Proportional (P) [68], proportional-derivative (PD) [100, 101], or proportional-integral-derivative (PID) [15, 71] controllers are widely implemented for flow and force regulation. Pulse width modulation (PWM) signals have been used to open and close solenoid valves [51] and can be implemented in many control strategies for different applications. Other kinds of controllers can be used depending on the system nature and on the task objective. For instance, nonlinear controllers, fuzzy approaches, or optimal linear control schemes could be developed for specific SEG systems. For instance, [102] provides an interesting review of soft robotic manipulator control strategies that could be considered to be applied in SEG systems.

SEG operation is based on force and position requirements to emulate human hand functions. These requirements, among others, are taken into account to define the control strategy to be synthesized. For example, SEG should have less than 10 minutes of setting time to become a useful tool for therapists [103]. Regarding fluid actuation, 10 N to 15 N are required for grasping tasks [11, 41]. SEG must be able to generate 7 N per finger or around 25 N on the whole hand with distributed forces along the fingers to minimize pressure location points, according to [34]. Normally, actuators with variable stiffness require 120 kPa for pinching and 160 kPa for grasping [18] while multisegment actuators require between 345 kPa and 400 kPa for flexion motion [51]. Desired joint ROM define positions to be reached by the patient when using a SEG system and provide reference variables to be controlled.

To evaluate SEG effectiveness in rehabilitation or assistance tasks, surface electromyography (EMG) has been implemented to detect user movement intentions [53], point out muscle contractions [16], control finger motion, and force level activation of muscles [90] since this is a noninvasive procedure that prevents muscle injuries.

During gripping tasks for finger flexion-extension, EMG signals are captured from the extensor digitorum communis (EDC) muscle together with the flexor digitorum superficialis (FDS) [20, 50] or with the flexor digitorum profundis (FDP) muscle [43, 73] since these muscles have been used and tested to work properly when implementing EMG procedures and due to the number of fingers they are connected with. Then, data obtained from a set of electrodes are amplified, filtered, quantified, and converted from analog to digital signals during SEG use [104]. This electrical stimulation should be monitored at least every 10 minutes to avoid muscle fatigue [103]. EMG signals can be used as control inputs when it is required to move specific hand joints that are connected to the aforementioned muscles. Due to stable behaviours, force myography (FMG) signals have been proposed to control the intention of the movement on SEG systems [20].

Motor impairment scales are applied to evaluate patient ROM to determine SEG operation ranges before starting an aided rehabilitation process. These scales serve for the evaluation of the damage that each patient has. According to [38, 105], patients with an Ashworth spasticity index (ASI) value less than or equal to three can use a soft exoglove. A modified Ashworth scale (MAS) value less than or equal to two defined the use of a soft exoglove for active flexion-extension, according to [106]. SEG operation is also based on the functional independence measure (FIM) of the patient by which the value goes from 1 to 7 depending on the assistance intensity [45]. Thus, for values above 3, patients present more autonomy [36].

#### 2.2.6. Assessment Criterion

To ensure patient safety and SEG operation, several tools such as joint contractions [31, 54], bending angles [71], 3D visual motion analysis [11], or optical ROM at specific joints [44] have been employed to evaluate SEG performance. Other methods have opted for using mathematical models together with the finite element method (FEM) for hand and finger trajectory characterization [67]. To assess patient satisfaction when using SEG systems, questionnaires have been considered [88].

SEG assessment can be also done based on the blocked [48], grasping [21], pinching [44], or fingertip [14] forces that are quantified using bottles, cups, balls, telephones, cans, or fruits with variable mass, size, and texture [44, 51]. For cylindrical objects, the diameters go from 50 mm to 120 mm [21, 75] with a mass of 300 g [107]. Experimental tests on SEG assessment have been carried out with dummy hands [71] and healthy individuals [44] or combining healthy people and stroke survivors [75]. Other SEG evaluations perform tasks with/without a soft exoglove and compare them [46, 92]. ROM data have been collected when using a soft exoglove and without it [31].

To assess hand function and ROM using SEG systems, patients undergo coordination and dexterity tests. For example, the Kapandji score is used to evaluate thumb performance on pinching and grasping tasks [108]. SEG assessment also considers the motricity index test (MIT) [105], the Fugl-Meyer assessment (FMA) [46], the nine-hole peg test (NHPT) [38], the Jebsen-Taylor hand test (JTT) [44], the box and block test (BBT) [11], the Purdue pegboard test (PPT) [45], or some writing tasks [109].

For each patient, one or more of the aforementioned methods could be chosen by his/her motor impairment or by the therapist in charge of the respective rehabilitation protocol in order to assess SEG systems.

Some authors have focused more on statistical analysis about user condition than SEG performance [5, 40]. They seek for a specific target group, rehabilitation time, training tools, age, or gender, for instance.

### 2.3. SEG Usability Criteria

To guarantee a friendly and comfortable SEG use, modularity, portability, customization, mode of intervention, and cost criteria must be considered to develop a soft exoglove with particular characteristics as easy to put on and operate, working in an intuitive way, and having low cost. These criteria are discussed below.

#### 2.3.1. Modularity Criterion

SEG designs have opted for modular configurations to ease donn and doff as in the cases of [21, 72, 83]. Connections can be assembled to work on targeted tasks, and actuators are mounted one by one [39]. Besides, modular designs for bending motions with deployable mechanisms have been adopted to reduce weight and allow natural motion [48]. SEG quality can be improved by a modularized system with relatively low cost customization, easy maintenance, and low power consumption [23]. Additionally, modular architectures allow for the replacement of feasible SEG components [89]. Based on this information, modularity is highly recommended as one of the main characteristics of SEG systems.

#### 2.3.2. Portability Criterion

To cope with patients' demands and to guarantee continuous rehabilitation protocols, the use of SEG outside clinics has become a main design concern to foster external rehabilitation [38, 110]. Nevertheless, to achieve this objective, SEG performance depends on the number of hours they can operate continuously without having a fixed power supply. According to [51], an effective soft exoglove should achieve, without problems, 2 hours of continuous operation or from 4 to 6 hours of intermittent operation.

Moreover, the runtime of batteries should be more than one hour in order to guarantee the development of a rehabilitation protocol session [100] until its completion or exert from 15 to 20 minutes of passive guidance [88]. Normally, lithium-polymer batteries are used since they can last 3.8 hours of continuous operation [23, 51].

Patients should take physical therapy sessions at rehabilitation facilities as well as at home [46, 92] in order to perform exercises on their own and not only depend on the availability of therapists [34]. SEG must be lightweight to allow their transportation [31, 73]. Thus, control unit boxes should be set up independently of the glove to minimize additional load [74]. Some power supply designs include waist belts [43, 51, 89], backpacks [73], boxes [11, 50], vests [84], waist pockets [53, 59], pockets [44], or a separate section located on another part of the human body [25, 31].

#### 2.3.3. Customization Criterion

As established by [18], conformability, adaptability, and customization are some features that can be taken into account to fit, properly, the hand of a patient and generate a compliant soft exoglove. Particularly, customization affects SEG operation since each finger length varies due to sex, age, and finger palm size [17]. Thus, fasteners [71] and Velcro® straps [8, 110] have been used to attach, conveniently, SEG to hands. Otherwise, deviations from a nonappropriate size or form may restrict hand movement or cause discomfort during SEG use [21].

#### 2.3.4. Mode of Intervention Criterion

To increase hand function rehabilitation, a bilateral mode in SEG systems results more beneficial than unilateral mode since patients can integrate healthy and paretic hand motions during rehabilitation therapy [75]. The bilateral mode is supported by a master-slave therapy concept where healthy limbs act as masters and soft devices as slaves [101]. Then, healthy limbs become a support for paretic limbs whereas devices working in the unilateral mode only exercise the impaired limb [111]. Bilateral mode rehabilitation could be recommended by the therapist as a function of the impairment. Then, SEG design could consider the mode of intervention depending on the associated rehabilitation protocol.

#### 2.3.5. Cost Criterion

It has been noted that researches are more interested in the functionality of their products than in their price, since only few works report SEG costs. However, SEG cost will determine one of the aspects for the success of an exoglove as a commercial product. Therefore, designers could generate low-cost readily available SEG systems. For instance, authors in [34] propose that the assembly should cost less than $30 USD in order to be a competitive choice. Another proposal establishes that manufactuing and electronics should be less than $200 USD [100]. According to [52], soft exosuits for the upper limb should cost less than $1000 USD, $465 USD for the elbow, and $470 USD for the hand. A detailed description about the component cost of these configurations could be found in [59]. SEG costs can vary due to the type of actuation, the type of components and materials, the weight, and the country where they were developed [112].

Remarkable results about cost analysis between conventional and aided therapy show that SEG rehabilitation is more affordable than therapist assistance since the reported cost associated with aided therapy is almost three times less expensive than the conventional one [45].

Currently, Neofect™, Glohera™, and Bioservo™ companies have already patented their systems which have been commercially exploited for hand rehabilitation and assistance. However, these commercial systems are available only in some countries and are, relatively, expensive. Importation and shipping costs must be added to final prices for countries and locations where these systems are not available.

## 3. SEG Design Guidelines

Based on the information provided in Section 2, Table 2 summarizes some of the main aspects related to the 13 proposed design criteria for SEG developments.

At present, SEG approaches are focused on improving functionality, strength, DOF, and ROM for object manipulation. Figure 2 and Table 3 provide information for each of the 91 SEG systems reviewed in this paper, associated with the following 15 aspects: (1) function: robot rehabilitation (RT) or assistance tasks (AT); (2) application: hand disability, stroke survivors, or SCI; (3) task: grasping, pinching, or manipulation; (4) palm design: OPD or CPD; (5) type and number of actuators: tendon-driven, pneumatic, or hydraulic; (6) assistance mode: PAM or AAM; (7) DOF per finger; (8) targeted fingers; (9) motions: flexion-extension, adduction-abduction, opponent, ulnar/radial deviations, and pronation-supination; (10) material; (11) weight; (12) force; (13) pressure; (14) control: CLC or OLC; and (15) assessment.

Figure 2 provides information related to the number of soft exogloves that have been developed in the last decade, being characterized by particular aspects. For example, the most important number of SEG systems that have been developed is focused on the passive assistance mode, CLC predominate over open-loop strategies, elastomers are preferred to other types of material, hydraulic actuation is not significant compared to the number of SEG devices using tendon-driven or pneumatic actuation, and SEG have been developed, mainly, to cope with stroke and hand disabilities as well as with rehabilitation and assistance problems.

Based on what has been presented so far, the following SEG design guidelines are highlighted in order to be considered when developing new SEG systems.

1. Rehabilitation and assistance tasks should be included in a single soft exoglove
2. SEG are primarily designed for stroke survivors and people with hand disabilities
3. Grasping is the main assistance task that has been addressed by SEG systems
4. SEG have been diversified for both OPD and CPD depending on the actuation
5. Tendon-driven and pneumatic are preferable types of actuators
6. AAM should be the priority motion guidance for SEG rehabilitation
7. Mostly SEG provide more than 10 DOF to reach hand motor function
8. A complete hand characterization must be included to tackle more DOF
9. All SEG provide, at least, flexion-extension motion. Furthermore, adduction-abduction and opponent motions are desirable
10. Elastomers have become the main material choice due to their flexibility, lightness, and adaptability
11. SEG systems should have a total mass of less than 200 g to enhance their efficiency
12. SEG should provide, at least, 5 N per finger to execute most of ADL
13. Regarding pneumatic actuation, SEG should work between 100 and 300 kPa
14. CLC controllers are preferable to OLC in order to ensure patient safety and system precision. Particularly, PD controllers have been mostly implemented
15. Fingertip forces, ROM, and EMG are the most used tools to evaluate SEG effectiveness

Table 3 provides detailed information related to the 15 aspects illustrated in Figure 2 for 91 devices that have been analysed in order to identify, classify, and discuss the 13 aforementioned criteria and to establish the previous 15 design guidelines for SEG systems. For example, the third system has eight DOF, focuses on grasping assistance tasks, has a closed palm configuration, is passively driven (CLC) by cables, and performs flexion/extension of 3 fingers.

From the previous information reported in this paper, five core SEG developers have been identified and have marked trends in the design of soft exoglove systems. Hong Kai Yap is the author with the highest number of SEG contributions (see Table 3, items 25-31).

**Figure 3:**
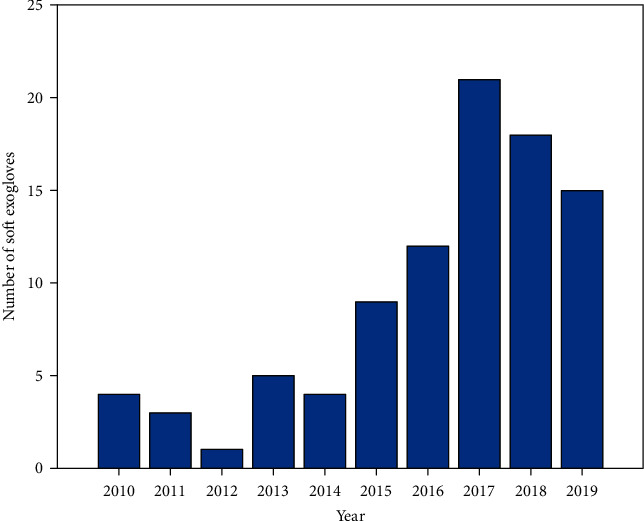
SEG developments in the last decade.

The number of SEG developments, from the last ten years, is plotted in Figure 3. According to literature, 2017 was the most productive year with 21 of the 91 contributions reported in this paper.

## 4. Discussion

In order to provide technical solutions for hand rehabilitation and assistance, multiple endeavours have been done during the last three years about SEG developments [128].

This review has identified areas of opportunity for the improvement of soft exogloves that are used in aided rehabilitation protocols and assistance tasks. Four main circumstances have motivated researchers to satisfy popular demand and increase SEG development since they represent an alternative and affordable approach to overcome hand disabilities. These circumstances are related to the increase in the number of people with hand motor deficits, to poor therapist availability, to the fact that clinical facilities are struggling to provide rehabilitation training, and to the expensive costs of these health services.

There are still significant challenges to face in soft exoglove design. For instance, power supply approaches are still limited and tendon-driven actuation necessitates motors without heating problems, whereas hysteresis issues should be solved in pneumatic systems to increase actuation cycles and durability along with lightweight and portable power supplies.

Regarding rehabilitation approaches, SEG systems must be endowed to exert intensive and repetitive routines without muscle fatigue and with minimal therapist assistance to excel above other rehabilitation options. SEG are a supportive aid that contributes to accelerated hand recovery by therapy protocols. Nevertheless, to achieve a desired rehabilitation task, an active contribution from the patient is required to regain strength, mobility, and ROM. Since the progress of each patient is variable, an AAM with time-triggered control could be implemented to regulate the input force of patients during rehabilitation processes, depending on their physical condition. SEG systems must encourage patient participation but do not execute all the rehabilitation work.

Several works have demonstrated that soft exogloves have the potential to offer safe human-robot rehabilitation or assistance. However, new trends show that these two tasks should be integrated into a unified system as it is reported by [46, 92]. To accomplish integral rehabilitation, SEG designers must consider that modular devices are expected to help therapists and patients depending on the impairment or on the rehabilitation protocol. This will be satisfied by connecting a soft exoglove device to a soft exosuit with a reliable and robust platform (see, for instance, [28]).

SEG shortcomings were identified concerning different hand sizes since most available systems are oriented towards adults. Thus, adjustable devices are recommended to have the possibility to initiate an early SEG-based rehabilitation program since this is a common advice given by therapists, no matter the dimensions of the patient's hand. So far, SEG systems are able to accomplish full open-close fist, grasping, lifting, and object release. Therefore, the systems reported in literature encompass from 8 to 14 DOF. Moreover, SEG characterization could be developed to obtain more DOF in order to expand the workspace if needed.

When soft exogloves are used, patient safety is a priority. Thus, human-machine interfaces with emergency buttons and haptic feedback must be considered for harmless interactions [35, 128] as stated in Section 2 of this paper, and several safety strategies must be incorporated in every SEG system. Moreover, SEG systems should not obstruct natural hand mobility and do not affect active ROM. Additionally, new developments are expected to provide patients and therapists with useful information in order to evaluate patient progress. Furthermore, the capability to automatically adjust the operation parameters as a function of the patient recovery level is desirable.

SEG self-manufacturing designs must ensure functional operation for home rehabilitation to provide low-cost systems. These considerations could allow to improve SEG features as hours of operation, power consumption, cleaning, and maintenance. Since Bluetooth communications have been considered between SEG systems and control interfaces [74], this or other communication systems must be part of new SEG devices when dealing with CLC strategies and for rehabilitation or assistance data analysis.

From this review, it can be pointed out that in recent years, the development of SEG has grown significantly in rehabilitation clinics and research groups. However, there is no comparison between research prototypes and those that have been already commercialized because the level of their technological maturity is different for each of them. Commercialized SEG systems must have evolved from research prototypes. The main difference between these two types of devices is the one related to their technological maturity. For instance, research prototypes can reach, in favorable cases, a technology readiness level (TRL) of 4 or 5 while commercialized products have the highest TRL of 9 in China [129, 130]. The evolution of a research prototype going from a 5 TRL to a certified product with 8 TRL and to a commercial product with a 9 TRL can take several years and require significant quantities of money. Moreover, medical devices having official approvals or certifications as that of the Food and Drug Administration (FDA) or the Conformité Européenne (CE) can be commercialized since they satisfy specific requirements and standards while research prototypes focus, mainly, on satisfying functional aspects. Then, it can be stated that commercialized medical devices are reliable due to the fact that they have completed the product design cycle reaching the product life-cycle management, while research prototypes have not begun the product development cycle or their industrial manufacture yet.

New-generation products should seek for an affordable trade-off between cost and benefit and include the possibility to perform assistance or rehabilitation therapy at home or in specialized clinics to ensure that rehabilitation protocols, defined by therapists, are efficiently executed.

SEG designs should provide acceptable appearance, comfort, and functionality to patients. Hence, it is highly recommended that SEG systems consider accessible technologies that could, additionally, create dynamic environments where patients can have pleasant therapy sessions. SEG require materials with appearance and elastic modulus similar to human tissues. Thus, smart polymers represent the primary current choice due to their biomimetic qualities to develop lightweight devices with modular OPD [128]. Besides, elastomers have been shown to be compliant wearable components with the ability to vary their form and increase the ROM based on the shape of the human hand.

Modularity plays a significant role when dealing with maintenance aspects of SEG systems as well as with costs and should be considered in new SEG developments. Besides, modularity can play a significant role when dealing with rehabilitation of different fingers or DOF. Regarding portability in new SEG developments, minimizing the dependence of energy sources becomes a challenge that must be addressed by researchers and engineers.

It has become clear that a SEG device that allows adaptation (customization) to a larger number of patients without the need for component replacements will be preferable to another system that only works for a certain size of hands.

## 5. Conclusions

Scientific and technical communications concerning wearable SEG for hand rehabilitation and assistance tasks applied to stroke survivors or people with hand disabilities have been extensively reviewed and reported in this paper. SEG design criteria have been identified, classified, and established into 2 function, 6 operation, and 5 usability criteria.

This paper also provides 15 guidelines for SEG design, a detailed description of 91 SEG that have been analysed based on the aforementioned criteria, and a discussion that considers different aspects in order to enhance future SEG developments.

From this review, it is highlighted that patient safety should be a priority characteristic during SEG operation, and then, it should be guaranteed in every new SEG development. This goal can be achieved by working closely with a therapist, as recommended in [28], as well as incorporating safety in mechanical and electronical parts and in the programming of the SEG device. Moreover, safety standards have been referenced to be considered in every SEG development.

It has been remarked that several efforts have been made in terms of SEG designs. However, there is still room to improve these devices. Then, this paper provides suggestions on patient safety, functional and continuous operation, friendly interaction, feedback information, and materials.

Other areas to be explored include hybrid SEG systems where new assembly techniques ensure force transmission or the use of electroencephalography signals to monitor brain activity when SEG rehabilitation is performed. SEG systems should be able to combine passive and active assistance modes along with bilateral training to enhance recovery processes and to encourage patients. The mentioned SEG design criteria provide perfectible guidelines to improve their performance and represent a basis to develop SEG robust designs.

## Figures and Tables

**Table 1:** Actuators for SEG systems.

Actuation	Types	Advantages	Disadvantages
Electrical	(i) Muscle wires (ii) Tendon-driven (iii) Shape memory alloys	(i) Cable paths reduce friction (ii) Provides continuous force (iii) Stores energy (iv) Commercial availability	(i) Complex transmissions (ii) Continuous hours of operation are restricted (iii) Nonlinearity of the system makes control difficult

Pneumatic	(i) FREA (ii) Inflatable chambers (iii) Pneumatic artificial muscles	(i) Allows multiple DOF (ii) Supports their structural shape (iii) Allows adaptability (iv) Lightweight	(i) Requires compressed air (ii) Requires a reservoir (iii) Inaccurate forces (iv) Problems with leaks (v) Portability is restricted

Hydraulic	(i) Fluid chambers	(i) High load capacity and power supply (ii) Low cost (iii) Allows multiple DOF	(i) Heavy systems (ii) Problems with leaks (iii) Portability is restricted (iv) Requires a reservoir and a pump

**Table 2:** Proposed criteria and considerations for SEG system design.

Type	Criteria	Considerations
Function	Rehabilitation	Stroke survivors and hand disabilities
	Assistance	Grasp, grab, pinch, lift, hold, and release tasks

Mobility	Actuation	Cable-driven, pneumatic, or hydraulic
	Materials	Fabric, synthetic leather, neoprene
	Motion guidance	Passive or active
	Manufacture	3D printing+other procedures
	Operation & control	45 minutes/day, OLC or CLC
	Assessment	7 N/finger or 10 to 15 N for 1 kg objects

Usability	Modularity	Open or closed palm configuration
	Portability	500 g or less than 3 kg for the whole system
	Customization	Right size, fasteners or Velcro® straps
	Mode of intervention	Bilateral or unilateral
	Costs	Assembly less than $30 USD

**Table 3:** Soft exoglove design criterion classification.

SEG # (Ref.)	Function	Application	Task	Palm design	Actuation (number)	Assistance mode	DOF/finger	Fingers	Motion	Material	Weight (g)	Force (N)	Pressure (kPa)	Control	Assessment
1 [14]	AT	Stroke survivors, SCI	Grasping	CPD	Tendon-driven (1)	PAM	8	Index, middle, thumb	Flexion-extension	Synthetic latex	80	18	—	CLC	Fingertip and blocked force
2 [17]	AT-RT	Hand disability	Grasping	CPD	Tendon-driven (3)	PAM	8	Index, middle, thumb	Flexion-extension	Synthetic latex	—	—	—	CLC (PI, PD)	EMG
3 [19]	AT	Stroke survivors, SCI	Grasping	CPD	Tendon-driven (3)	PAM	8	Index, middle, thumb	Flexion-extension	Synthetic latex	194	20	—	CLC	EMG
4 [21]	AT	Hand disability	Grasping	OPD	Tendon-driven (2)	PAM	8	Index, middle, thumb	Flexion-extension	Silicone KE-1300	—	20	—	—	EMG
5 [23]	AT	Hand disability	Grasping, pinching	OPD	Pneumatic (4)	PAM	11	Index, middle, thumb	Flexion-extension, adduction-abduction	Silicone KE-1300 T	350	22.5	300	CLC	ROM, grip strength
6 [22]	AT	SCI	Grasping, pinching	OPD	Tendon-driven (3)	PAM	9	Index, middle, thumb	Flexion-extension, opponent	Silicone KE-1300 T	104	10.3	—	CLC	Grip strength
7 [8]	RT	Hand disability	Grasping, pinching	OPD	Pneumatic elastomer (4)	PAM	14	All except thumb	Flexion-extension, curl	Neoprene	160	—	—	CLC	FEM analysis
8 [51]	AT-RT	Hand disability	Grasping, pinching	OPD	Hydraulic elastomer (2)	PAM	15	All	Flexion-extension, opponent	Neoprene	—	—	400	CLC	ROM, EMG
9 [11]	AT-RT	SCI	Grasping	OPD	Hydraulic elastomer (2)	PAM	15	All	Flexion-extension, opponent	Textile	—	10-15	—	CLC	ROM, BBT
10 [50]	AT	Hand disability	Grasping, holding	OPD	Hydraulic elastomers	PAM	15	All	Flexion-extension, opponent	Elastomer	—	14	413	CLC	ROM, EMG
11 [36]	RT	Stroke survivors	Gripping	OPD	Pneumatic	PAM	16	All	Flexion-extension, opponent, adduction-abduction	—	—	—	—	—	ROM: MI, BBT, FIM
12 [105]	AT-RT	Hemiplegic patients	Gripping, pinching	OPD	Pneumatic	PAM	16	All	Flexion-extension, opponent, adduction-abduction	—	—	—	—	—	ROM: MI, NHPT test
13 [45]	AT-RT	Stroke, SCI	Gripping, pinching	OPD	Pneumatic	AAM-PAM	16	All	Flexion-extension, opponent, adduction-abduction	—	—	—	—	—	ROM: NHPT, FIM test
14 [113]	RT	Stroke, SCI	Gripping, pinching	OPD	Pneumatic	AAM-PAM	15	All	Flexion-extension, opponent, adduction-abduction	—	—	—	—	—	ROM: MI, NHPT test
15 [38]	RT	Stroke survivors	Gripping	OPD	Electrical (5)	PAM	15	All, wrist	Flexion-extension, opponent, adduction-abduction	—	—	—	—	—	Ashworth index
16 [114]	AT	—	Grasping	CPD	Hydraulic	PAM	14	All	Flexion-extension	—	2620	12	550	CLC	Pressure regulating
17 [39]	RT	Stroke survivors, SCI	Grasping, manipulation	OPD	Tendon-driven	AAM-PAM	14	All, wrist, forearm	Flexion-extension, opponent, pronation-supination	—	—	—	—	—	ROM: FMA, JTT, PPT
18 [115]	RT	Stroke survivors	Grasping, manipulation	OPD	Tendon-driven	AAM	14	All, wrist, forearm	Flexion-extension, opponent, pronation-supination	—	—	—	—	—	—
19 [47]	RT	Stroke survivors, SCI	Grasping, manipulation	OPD	Tendon-driven	AAM-PAM	14	All, wrist, forearm	Flexion-extension, radial-ulnardeviations, pronation-supination	Elastomer	132	—	—	CLC	ROM
20 [73]	AT-RT	Stroke survivors, SCI	Grasping, pinching	CPD	Tendon-driven (5)	AAM-PAM	12	All	Flexion-extension	Lycra, fabrics	—	15	—	CLC	Grip force
21 [84]	AT	Hand disability	Grasping, pinching	CPD	Tendon-driven (3)	PAM	8	Middle, ring, thumb	Flexion-extension	Synthetic leather	700	20	—	CLC (PID)	Grasping power test
22 [85]	AT-RT	Hand disability	Grasping	OPD	Tendon-driven (5)	PAM	15	All	Flexion-extension, adduction-abduction	Silicone rubber	—	17.25	165	CLC	ROM: tactile pressure
23 [116]	RT	Hand disability	Grasping	OPD	Tendon-driven (5)	PAM	15	All	Flexion-extension, adduction-abduction	Elastomer	—	—	—	—	—
24 [24]	AT	Material handling	Grasping	CPD	Tendon-driven	PAM	—	All	—	Fabrics	—	—	—	CLC	Lift forces
25 [18]	AT-RT	Hand disability	Grasping, pinching	CPD	Pneumatic (5)	AAM	14	All	Flexion-extension	Fabrics	200	—	160	CLC	ROM
26 [41]	RT	Stroke survivors	Grasping	CPD	Pneumatic (4)	PAM	12	All except thumb	Flexion-extension	Fabrics	200	9.25	200	CLC	fMRI, optical ROM
27 [43]	AT	Grasp pathologies	Grasping, releasing	CPD	Pneumatic (5)	PAM	15	All	Flexion-extension, opponent	Lycra, fabrics	170	13.6	153	CLC	Optical ROM, EMG
28 [1]	AT-RT	Stroke survivors	Grasping, releasing	OPD	Pneumatic (5)	AAM	14	All	Flexion-extension	Neoprene	150	—	100	CLC	ROM, torque
29 [106]	AT-RT	Stroke survivors	Grasping, pinching	OPD	Pneumatic (4)	PAM	12	All except thumb	Flexion-extension	Lycra, fabrics	180	10.2	120	CLC	fMRI, optical ROM
30 [44]	AT	Stroke survivors	Grasping, lifting, releasing	OPD	Pneumatic (5)	AAM-PAM	14	All	Flexion-extension	Lycra, fabrics	180	12-36	120	CLC	ROM, EMG
31 [75]	AT-RT	Hand disability	Grasping, manipulation	CPD	Pneumatic (5)	AAM	14	All	Flexion-extension	Fabrics	99	13.6	275-375	CLC	Gripping force
32 [53]	AT	Hand disability	Grasping	CPD	Tendon-driven (1)	PAM	8	Index, middle, thumb	Flexion-extension	Synthetic latex	500	10	—	CLC	ROM, gripping force
33 [59]	AT	Muscle weakness	Grasping	CPD	Tendon-driven (2)	PAM	8	Index, middle, thumb, elbow	Flexion-extension	Neoprene	1200	10	—	CLC (PD)	ROM, gripping force
34 [52]	AT	Hand disability	Grasping	CPD	Tendon-driven (3)	PAM	8	Index, middle, thumb	Flexion-extension	Neoprene	500	—	—	CLC (PD)	ROM, gripping force
35 [25]	AT-RT	Heavy tasks	Grasping holding	CPD	Tendon-driven (3)	PAM	14	All	Flexion-extension	Fabrics	770	—	—	CLC	Grasping force
36 [30]	AT-RT	Hand disability	Grasping, manipulation	OPD	Pneumatic, hybrid (4)	PAM	12	All except thumb	Flexion-extension	Nylon	150	2.5	230	CLC	Bending forces, EMG
37 [48]	RT	Hand disability	Grasping, manipulation	CPD	Pneumatic, hybrid (5)	PAM	14	All	Flexion-extension	—	—	—	165.4	CLC	ROM, joint angles
38 [42]	RT	Hand pathologies	Grasping	OPD	Pneumatic elastomers (5)	PAM	14	All	Flexion-extension	Textile	—	—	526	CLC	ROM, fatigue test
39 [16]	AT	Hand disability	Grasping, pinching	CPD	Pneumatic (5)	PAM	15	All	Flexion-extension, opponent	Synthetic leather	135	9	200	CLC	ROM, EMG
40 [15]	RT	Stroke survivors	Grasping, releasing, pinching	CPD	Pneumatic (5)	PAM	14	All	Flexion-extension	Lycra	—	15	60	CLC (PID)	ROM: FMA, BBT
41 [31]	RT	Hand disability	Grasping	CPD	Tendon-driven (3)	PAM	8	Index, middle, thumb	Flexion-extension	Synthetic leather	—	—	—	CLC	ROM, joint angles
42 [35]	AT-RT	Hand paralysis	Grasping	CPD	Tendon-driven (1)	PAM	8	Index, middle, thumb	Flexion-extension	Polyester fiber	50	35	—	CLC	ROM, EMG
43 [117]	AT-RT	Hand disability	Gripping	CPD	Tendon-driven (3)	PAM	8	Index, middle, thumb	Flexion-extension	Fabrics	—	—	—	CLC	ROM, gripping force
44 [118]	AT	Older adults	Gripping	CPD	Tendon-driven (3)	PAM	8	Index, middle, thumb	Flexion-extension	Fabrics	85	—	—	CLC	Pinch strength, JTHFT
45 [46]	AT-RT	Hand disability	Gripping	CPD	Tendon-driven (5)	PAM	14	All	Flexion-extension	—	—	—	—	CLC	Pinching force, JTHFT test
46 [54]	RT	Hand disability	Grasping	CPD	Tendon-driven (5)	PAM	14	All	Flexion-extension	Fabrics	—	—	—	CLC	ROM, joint angles
47 [71]	RT	Stroke survivors	Grasping	OPD	Pneumatic (5)	PAM	16	All	Flexion-extension, opponent, adduction-abduction	Nylon	—	20	—	CLC (PID)	ROM, joint angles
48 [34]	RT	Stroke survivors	Grasping	CPD	Spring mechanism (5)	PAM	14	All	Flexion-extension	Synthetic leather	200	22.59	—	—	Bending force
49 [110]	RT	Stroke survivors	Grasping	OPD	Pneumatic (5)	PAM	14	All	Flexion-extension	—	—	—	—	CLC	Electroencephalography
50 [89]	AT-RT	Stroke survivors	Gripping	OPD	Pneumatic (5)	PAM	14	All	Flexion-extension	Elastomer	—	41.8	200	CLC	EMG, ROM, gripping force
51 [107]	AT	Hand disability	Grasping, manipulation	CPD	Cable-driven (4)	PAM	11	All except little	Flexion-extension	—	250	16	—	CLC	ROM, pinching force
52 [67]	RT	Hand disability	Grasping	CPD	Pneumatic (4)	PAM	12	All except thumb	Flexion-extension	Nylon	—	3	200	—	FEM & ROM
53 [119]	AT	Hand disability	Grasping, manipulation	OPD	Pneumatic artificial muscles (5)	PAM	14	All	Flexion-extension	Fabrics	161	10	200	—	FEM, fingertip force
54 [120]	AT-RT	Older adults	Grasping, manipulation	OPD	Tendon-driven (5)	PAM	14	All	Flexion-extension	TPU, NINJAFLEX™	50	40	60	CLC	Pressure regulation & fingertip force
55 [74]	AT-RT	Hand disability	Grasping, pinching	OPD	Tendon-driven (5)	PAM	14	All	Flexion-extension	TPU	330	22 pinch, 48 grasp	—	CLC	Pinching and grasping forces
56 [55]	AT-RT	Hand disability	Grasping	CPD	Shape memory alloys (5)	PAM	14	All	Flexion-extension	Fabrics	—	40	—	CLC	ROM, fingertip-tendon force
57 [4]	AT	Stroke survivors	Grasping, manipulation	OCP	Tendon-driven (5)	PAM	12	All except thumb	Flexion-extension	Polymer	340	—	—	CLC	Gripping force
58 [112]	AT	CP, stroke survivors	Grasping	CPD	Tendon-driven (3)	PAM	8	Thumb, index, and middle	Flexion-extension	Synthetic leather	55	48	—	CLC	Grasping and fingertip forces
59 [121]	AT	Supportive aid	Grasping	CPD	Shape memory alloys (5)	PAM	12	All except little	Flexion-extension, radial abduction, palmar abduction, opposition	Synthetic leather	85.03	11	—	CLC	Grasping force, ROM
60 [122]	RT	Stroke survivors	Grasping	OPD	Pneumatic (1)	PAM	3	Index	Flexion-extension	Ecoflex™ 00-30	—	1.17	30	CLC	Bending angle
61 [13]	AT	Older adults, hand disability	Grasping, pinching	CPD	Pneumatic artificial muscles (5)	PAM	14	All	Flexion-extension	Rubber	—	5.7-14, 20-25	500	CLC	Grasping and pinching forces
62 [109]	AT	SCI	Grasping	CPD	Tendon-driven (3)	PAM	8	Thumb, index, and middle	Flexion-extension	—	—	7.39	—	CLC	Writing tasks, grasping force
63 [7]	AT-RT	Stroke survivors	Grasping	CPD	Pneumatic, tendon-driven (5)	PAM	14	All	Flexion-extension	Nylon	—	5	300	CLC	Ashworth test
64 [123]	RT	Stroke survivors	Grasping	CPD	Tendon-driven (4)	PAM	12	All except thumb	Flexion-extension	—	—	—	—	CLC	FMA test
65 [82]	RT	Hand disability	Grasping	OPD	Pneumatic FREAs (5)	PAM	14	All	Flexion-extension	Nylon	280	11.27	250	CLC	Grasping test FEM
66 [108]	RT	Hand disability	Grasping	OPD	Pneumatic FREAs (1)	PAM	1	Thumb	Opposition	Elastomer	586	—	150	CLC	Kapandji test
67 [124]	AT	Stroke survivors	Grasping	OPD	Pneumatic FREAs (5)	PAM	14	All	Flexion-extension	Silicone rubber	207	—	200	CLC	Bending force FEM
68 [125]	AT	SCI	Grasping, manipulation	OPD	Pneumatic (5)	PAM	14	All	Flexion-extension	Fabric	77	15	172	CLC	Lifting force
69 [99]	AT	Stroke survivors	Grasping	CPD	Electrical	PAM	15	All, wrist	Flexion-extension	Neoprene fabric	—	—	—	CLC	BBT test
70 [98]	RT	Stroke survivors	Grasping	CPD	Tendon-driven (1)	PAM	3	Index, wrist	Flexion-extension	Lycra	—	—	—	CLC	ROM
71 [61]	RT	Kinesthetics, haptic feedbacks	Pressing	OPD	Pneumatic (2)	PAM	6	Index and middle	Flexion	Silicone rubber	—	16.66	210	CLC	Virtual reality haptic feedback
72 [126]	AT	Hand disabilities	Grasping, holding	CPD	Pneumatic (4)	PAM	15	All	Flexion-extension, opposition	—	65	—	—	CLC	Grabbing force
73 [57]	AT	Hand disabilities	Grasping	CPD	Pneumatic (4)	PAM	11	All except little	Flexion-extension	—	160	25	500	CLC	Bending angle
74 [127]	AT	Hand disabilities	Grasping	CPD	Pneumatic (5)	PAM	14	All	Flexion-extension	Elastomer	180	3	300	CLC	Grasping force
75 [68]	RT	Stroke survivors	Grasping	CPD	Pneumatic (5)	PAM	14	All	Flexion-extension	Latex	—	—	—	CLC proportional	Bending angle
76 [72]	RT	Stroke survivors	Grasping	OPD	Tendon-driven (5)	PAM	9	Thumb, index, and middle	Flexion-extension, opposition/reposition	Silicone KE-1300 T	120	12	—	CLC	Bending angle
77 [49]	AT	Stroke survivors	Grasping	CPD	Tendon-driven (5)	PAM	12	All except thumb	Flexion-extension	Lycra	—	16-17	—	CLC	Fingertip force ROM
78 [100]	RT	Stroke survivors	Grasping	CPD	Tendon-driven (5)	AAM-PAM	14	All	Flexion-extension	Elastomer	>1000	—	—	CLC (PD)	Fingertip force ROM
79 [69]	AT-RT	Hand disabilities	Grasping	CPD	Pneumatic FREA (5)	PAM	16	All	Flexion-extension, opponent, adduction-abduction	Polyester	76	0.8	150	CLC	Bending angle and force output
80 [88]	RT	Stroke survivors	Grasping	OPD	Pneumatic (5)	PAM	14	All	Flexion-extension	RTV-4234T4, silicon	—	—	50	CLC (PD)	Bending angle
81 [40]	RT	Stroke survivors	Grasping	CPD	Tendon-driven (5)	PAM	14	All	Flexion-extension	Fabrics	—	—	—	—	Virtual reality, FMA
82 [10]	AT	Hand disabilities	Grasping	Semiopen	Motor-tendon (5)	PAM	14	All	Flexion-extension	Cotton fabric	600	—	—	On-off control	Grasping force output
83 [90]	AT	Hand disabilities	Grasping	CPD	Tendon-driven (5)	PAM	12	All except little	Flexion-extension, opponent	—	220	83	—	CLC	Grasping force ROM
84 [26]	AT	Heavy tasks	Manipulation tasks	CPD	Tendon-driven SMA (5)	PAM	14	All	Flexion-extension	Rubber	—	70	—	CLC (PID)	Force output
85 [104]	AT	Hand disabilities	Grasping, releasing	CPD	Pneumatic (5)	PAM	14	All	Flexion-extension	Fabric	160	88.29	180	—	EMG signals
86 [91]	RT	Hand disabilities	Grasping	CPD	Steel spring	AAM-PAM	14	All	Flexion-extension	—	401	30.87	—	CLC	ROM, force output, EEG signals
87 [58]	RT	Hand disabilities	Grasping	CPD	Pneumatic (5)	PAM	14	All	Flexion-extension	—	150	40	300	OLC	EMG signals, grasping forces
88 [96]	AT	Stroke survivors	Grasping	CPD	Tendon-driven (5)	AAM-PAM	10	All	Flexion-extension	Nylon	258	—	—	OLC	EMG signals, grasping and lifting forces
89 [103]	RT	Stroke survivors	Grasping	CPD	Tendon-driven SMA (5)	PAM	14	All	Flexion-extension	—	—	—	—	—	Teleoperation, time output
90 [101]	RT	Stroke survivors	Grasping	CPD	Pneumatic (5)	PAM	14	All	Flexion-extension	RTV-4234T4, silicon	—	—	105	CLC (PD)	Finger trajectories and angle
91 [20]	AT	Hand disabilities	Grasping	CPD	Tendon-driven (5)	PAM	14	All	Flexion-extension	Polyester and neoprene	—	—	—	CLC	Griping force and FMG signals

## Abbreviations

AAM: Active assistance mode
ADL: Activities of daily living
ARM: Active resistive mode
ASI: Ashworth spasticity index
AT: Assistance tasks
BBT: Box and block test
CLC: Closed-loop control
CE: Conformité Européenne
CMC: Carpometacarpal
CP: Cerebral palsy
CPD: Closed palm design
DOF: Degrees of freedom
EMG: Electromyography
FDA: Food and Drug Administration
FEM: Finite element method
FIM: Functional independence measure
FMA: Fugl-Meyer assessment
FMG: Force myography
FREA: Fiber reinforced elastomer actuators
GUI: Graphical user interface
IP: Interphalangeal
JTT: Jebsen-Taylor hand test
MAS: Modified Ashworth scale
MCP: Metacarpophalangeal
MIT: Motricity index test
NHPT: Nine-hole peg test
OLC: Open-loop control
OPD: Open palm design
PAM: Passive assistance mode
PD: Proportional-derivative
PID: Proportional-integral-derivative
PT: Purdue pegboard test
ROM: Range of motion
RT: Robot rehabilitation
SCI: Spinal cord injury
SEG: Soft exogloves
SMA: Shape memory alloys
RTV: Room-temperature-vulcanizing
TPU: Thermoplastic polyurethane
TRL: Technology readiness level.
